# Determining application rates of FYM and pressmud to improve soil health properties in salt degraded soils

**DOI:** 10.1371/journal.pone.0317463

**Published:** 2025-01-31

**Authors:** M. L. Dotaniya, M. D. Meena, R. L. Choudhary, M. K. Meena, V. D. Meena, H. V. Singh, R. S. Jat, R. K. Doutaniya, Kuldeep Kumar, Harpreet Singh, P. K. Rai

**Affiliations:** 1 ICAR-Indian Institute of Rapeseed-Mustard Research, Bharatpur, Rajasthan, India; 2 Department of Agronomy, SKN College of Agriculture, Jobner, Rajasthan, India; 3 ICAR-Indian Institute of Soil and Water Conservation, Research Centre, Kota, Rajasthan, India; 4 Regional Research Station, PAU, Gurdaspur, Punjab, India; ICAR - IIFSR: ICAR - Indian Institute of Farming Systems Research, INDIA

## Abstract

Improving food grain production by enhancing plant nutrient availability is critical for meeting future production. In this line, degraded soils may have the potential to meet the food demand of future population. However, the key challenge is excessive concentration of salts. It adversely mediates the soil fertility parameters, physical properties and soil enzymatic activities. Addition of organic substances, such as farm yard manure (FYM) and pressmud (PM), may improve soil health parameters. An incubation experiment was conducted with graded application of FYM (0, 2.5, 5 and 10 t/ha) and PM (0, 2.5, 5 and 10 t/ha) to monitor the nutrient release pattern of FYM and PM; and its effect on soil physico-chemical properties and soil enzymatic activities. The results showed that soil pH and EC were reduced after the one year incubation period. It was also observed that available plant nutrients like N, P, K, S and soil enzymatic activities reported highest in treatment FYM and PM (each applied 10 t/ha) over control. The findings of this study suggested that organic amendments can improve soil fertility, mitigate salt ion toxicity, and enhance food production potential, particularly in arid and semi-arid soils.

## Introduction

Salinity is a limiting factor in most of the part of the arid and semi regions of the globe. According to FAO [1], soil salinization affects approximately one-fifth of cultivated farmland globally, leading to severe reductions in agricultural output and endangering global food security. It was observed that the geogenic contribution of salt has been extended by the anthropogenic activities particularly over irrigation, addition of poor inputs, faulty crop management practices etc. Availability of essential plant nutrients are reduced due to higher concentration of calcium (Ca^2+^), magnesium (Mg^2+^), chloride (Cl^−^), sulphate (SO_4_^2−^) ions; and also sodium (Na), carbonate and bicarbonets in the soil [2]. Soil salinization and alkalization pose significant obstacles to global crop productivity especially in arid and semi-arid regions. Salt in the soil poses a significant threat to global food security, exacerbated by climate change. Salinity of the soil is a prevalent concern for numerous nations globally, especially those with semi-arid and arid climates. Soil salinity can disrupt various chemical and physical activities in the soil, including microbial processes crucial for soil fertility, productivity, and health [3]. Moreover, the physiological, biochemical, and genetic traits of cultivated plants can suffer negative effects due to soil salt content [4]. Salinized soils expose crops to multiple stressors, primarily ionic, osmotic, and oxidative stresses [5]. To survive in such conditions, plants require effective tolerance mechanisms that regulate ionic balance, maintain osmotic stability, and manage reactive oxygen species (ROS) to alleviate these adverse effects [6]. Understanding the chemistry of salts in soil, alongwith their dynamics, physico-chemical, and biogeochemical traits, is crucial [7]. Ionic and osmotic influences in soil can significantly impact the microbial communities, composition, and enzyme activity within the rhizosphere [8].

In most of the above limitation in saline soils, scientific management of abiotic stress should be addressed properly for enhancing the crop productivity. The population growth rate of India is increasing 1.67 billion by the year 2050 and needs approximately 400 million tonne (mt) food grain [9]. It is a challenge for researcher and agriculture management systems to feed growing population on 2.4 percent agriculture land. However, varietal development of crops, enhanced the production potential of crops, but plant nutrient availability hampered by the abiotic stress minimized the production in saline and alkaline soils [10]. Most of the crop production potential is directly related to the soil fertility might be maintained by addition of chemical fertilizers. However, addition of organic and inorganic substances improved the nutrient availability under saline soils. However, the amount and type of organic matter mediated the plant nutrient dynamics in soil and transformed into crop yield and soils health [11]. It was also observed that incorporation of organic substances improved the soil health parameters by managing the salt ion concentrations. Shortage of farm yard manure (FYM) application will be meet out by the huge amount of sugarcane industrial waste like bagasse, pressmud (PM). These are having significant amount of plant nutrients and organic carbon. Dotaniya et al. (2023) reported that addition of 10 t/ha organic substances (FYM and PM) improved the soil fertility parameters. Theses organic sources of plant nutrients are most effective as compared to the chemical fertilizers.

The sugarcane industry generates substantial quantities of pressmud, posing a significant disposal challenge. The residue remaining after filtering sugarcane juice is termed sugarcane pressmud [12]. Throughout the purification process, the juice is separated into a clear component, rising to the top for sugar production, and a mud sediment that accumulates at the bottom [13]. Often, PM is incinerated in brick kilns, resulting in the loss of millions of tons of valuable nutrients that could enrich the ecosystem [14]. Typically utilized as fertilizer, pressmud undergoes various methods to restore its fertilizing properties [15]. Most commonly, this industrial waste finds application in the production of wax, soil conditioner, and fertilizer. According to Bhosale et al. [16], applying sugarcane PM is cost-effective and offers excellent water retention, mulching properties, and a slow release of nutrients and trace elements.

Moreover, the augmentation of soil moisture, aggregate stability, and porosity is achievable. The management of agricultural residue return can alleviate the detrimental effects of allelo-chemicals on crop growth [17]. However, the impact of crop residues on heavy metals and soil pH is variable under different soil ecosystems [18]. In certain scenarios, crop residues can hinder the presence of specific heavy metals. Additionally, they aid in ameliorating saline-alkaline soils, reducing the bioavailability of various soil organic contaminants, and mitigating several soilborne diseases. A well-thought-out strategy for returning crop residues is imperative to enhance soil health. Initially, aligning the injection of nutrients from organic crop residues with the crop’s requirements is crucial [19]. Secondly, the decomposition of agricultural residues can be accelerated by combining crop residue return with partial nitrogen fertilizer, straw ripening agent, and lime, thereby enhancing the activities of soil microorganisms [19]. Thirdly, the breakdown of crop residues may be influenced by soil properties, climate conditions, and the quality of the residues themselves [20]. To uphold soil health, it is essential to establish a systematic theory for returning crop residues. Addition of FYM and PM improved the soil health parameters like improving soil aggregation and reducing compaction, enhances soil’s ability to retain water, provides essential nutrients, increases the soil’s ability to hold and exchange nutrients and directly improved the soil microbial process efficiency [21]. Application of crop residues can be advantageous for saline-alkaline soils by aiding in the regulation of salt and water levels [22]. It will help to improve the soil health properties and better soil conditions for plant growth. Soil enzymes are a useful indicator of soil quality for selecting organic material amendments [23]. The use of integrated nutrition management (INM) combined with organic manures, including press mud, urban compost, maize residue, green manures, and chicken manure, has been shown to enhance the activity of arylsulfatase, alkaline phosphatase enzymes in various cropping systems [24].

Despite extensive studies on FYM applications, limited information exists on the graded application of FYM and PM and their effects on nutrient availability patterns in salt degraded soils. In this backdrop, a incubation experiment was conducted to see the mineralization pattern of FYM and PM and their effect on plant nutrients and soil enzymatic activities under salt degraded soils.

## Materials and methods

### Location

Experiment was conducted at ICAR-Indian Institute of Rapeseed-Mustard Research (ICAR-IIRMR), Bharatpur, India located on 77.30° E longitude; 27.15° N latitude and the 178.37 meter mean sea level. It is under the crown of Indian Council of Agricultural Research, Ministry of Agriculture and Farmers Welfare, GOI. It is established basic and applied research in the field of rapeseed-mustard. According to the Government of India’s classification, the region belongs to agro-ecological zone III-B, with a mean annual temperature of 26°C and an average rainfall of 798 mm per year.

### Treatment details

To conduct the experiment, bulk saline soil samples were collected from the agricultural field of Deeg district near to Bharatpur, India. Samples were processed and sieved through 2 mm sieve, and kept in plastic container for further analysis. Graded levels of FYM (0, 2.5, 5 and 10 t/ha) and PM (0, 2.5, 5 and 10 t/ha) were applied in processed pot soils. Plastic pots, with a dimensions (28 cm × 21 cm × 17 cm) and designed for optimal aeration, were filled with 5 kg of collected soil. Prior to apply the organic substances in soil different plant nutrient composition was measured to compute the nutrient balancing among the treatments. The FYM was sourced from the research farm of ICAR-IIRMR in Bharatpur; while, the PM was obtained from the Daurala Sugarmill in Meerut, India. Standard analytical procedures were used to examine the properties of the PM and FYM. The results showed that the PM had a pH of 8.31, EC of 3.22 dS/m, potassium concentration of 0.037%, organic carbon (OC) of 9.54 mg/kg, total N of 0.019%, total P of 0.012 mg/kg, and sulfur (S) of 0.059%. In contrast, the FYM exhibited a pH of 7.24, EC of 1.63 dS/m, potassium concentration of 0.042%, OC of 14.09 mg/kg, total N of 0.54%, total P of 0.023 mg/kg, and S of 0.012%. Total 16 combination were made and conducted in 3 replicate designed. After thoroughly mixed soil and treatments were kept in small plastic containers by maintaining water at field capacity at laboratory temperature.

### Soil sampling and analytic procedure

Destructive soil samples were taken at 3, 6, 9 and 12 months time interval after the organic substances incorporated. After that, thoroughly processed and measured the physio-chemical properties of soils as initially sample collection. Soil pH (2:1 soil: CaCl_2_ ratio) and electrical conductivity (EC) was measured with the help of glass electrodes. However, prior to measure the parameters, pH meter was calibrated at pH 4, 7, 9.2 and EC meter by 0.5 *M* KCl solution at 25°C temperature. Walkley & Black carbon was measured with the help of the method mentioned in Singh et al. [25]. In this method 1 g processed sample was taken in conical flask and add 10 mL of 1 *N* K_2_Cr_2_O_7_ and 20 ml concentrated H_2_SO_4_ for initiated the chemical reactions. After that, slowly add the 200 ml distilled water and 10 ml orthophospharic acid; and 1 ml diphenylamine indicator to precisely observe the end point. Further, 0.5 *N* ferrous ammonium sulphate solution was taken in burette and back titrate till the green color appeared. Available N was measured by N-auto analysis system as mentioned the procedure by Subbiah and Asija [26]. For this 20 g soil samples mineralized by KMnO_4_ and liberated ammonia was captured in 2 percent boric acid. Again it was back titrated with H_2_SO_4_. Available P was measured by Olsen et al. [27] by using the 0.5 *M* NaHCO_3_. The potassium concentration in soil was measured as described in Hanway and Heidel [28] by 1 *N* ammonium acetate solution and shaking for 5 minutes. It represent the water and exchangiable concentration of K in solution and estimated through Flame photometer. The CaCl_2_-extractable S was also estimated by developing the BaCl_2_ turbidity and measured the intensity in spectrophotometer at 340 nm [29].

### Microbial parameter analysis

However, soil microbial activity was also measured to check the mineralization kinetic of plant nutrients and soil biomass carbon. In this experiment, alkaline phosphate, arylsulphate activities and DHA were measured at laboratory temperature (25°C). According to the procedure outlined by Tabatabai and Bremner [30], alkaline phosphatase activity in the soil was evaluated by measuring the amount of p-nitrophenol released, with the yellow coloration’s absorbance being recorded at 440 nm using a spectrophotometer. Dehydrogenase activity (DHA) was assessed using the procedure detailed by Casida et al. [31]. The method is based on the reduction of a colorless substrate, 2,3,5-triphenyltetrazolium chloride (TTC), by dehydrogenase enzymes in soil to form a red-colored compound, triphenyl formazan (TPF). The amount of TPF produced is directly proportional to the dehydrogenase activity in the soil. The TPF is then extracted using an ethanol; and quantified by measuring its absorbance using a spectrophotometer at 485 nm. Arylsulphatase activities were determined based on the approach described by Tabatabai and Bremner [32]. Arylsulphatase activity in soil is determined by measuring the release of p-nitrophenol (p-NP) from p-nitrophenyl sulfate (p-NPS) during enzymatic hydrolysis. The p-nitrophenol released reacts to form a yellow color that is quantified spectrophotometrically at 400 nm.

### Statistical analysis

Treatment screening incubation experiment was conducted in complete randomized design (CRD) with three replications. Total 16 treatment combinations and their interaction were also calculated and compared the significance of treatment at 5 percent level of significance (p <  0.05) as mentioned in Gomez and Gomez [33].

## Result and discussion

### Effect on soil pH and EC

After the one year experiment, collected soil samples were analysed for soil pH and EC and compared for significant at 5 percent level of significance. It was observed that throughout the incubation period soil pH fluctuate more and reduced upto 7.87 in treatment F_10_PM_2.__5_ and maximum in control treatment. However, all the treatments are statistically non significant at p <  0.05 in after 3 months time interval. The trend showed that the addition of organic substances upto 10 t/ha through FYM and PM reduced the soil pH. The analysis trend of soil pH after 3 months time interval showed significant reduction of pH by the application of FYM and PM alone as well as in combinations. At 6 month time interval, soil pH reduced from 8.22 to 7.75 in FYM applied at 10 t/ha alone. Similar way, again lowest pH (7.75) was reported in FYM treated plot at 10 t/ha at 9 month time interval. After the one year incubation period, soil pH was measured and reported that addition of PM upto 10 t/ha didn’t affect the PH value, however after this, significantly reduced the pH value. In case of FYM, 2.5 t/ha application reduced soil pH from 8.24 to 7.83; whereas, further enhancement upto 5 t/ha did not affect the value, application upto 10 t/ha non significantly reduced the soil pH. Electrical conductivity of the soil were also mediated by the application of FYM and PM. Initially, EC was 0.573 dS/m and by the adding of PM it was little bit enhanced; however, addition of FYM it was significantly reduced upto 0.517 dS/m in 5 t/ha treatment (equal amount of FYM and PM) at 3 month time interval. However, similar trend was observed at 9 and 12 months of time interval. In most of the treatments, FYM treatments drastically reduced the soil EC more than the FYM. The combined observation among the time period and treatments showed that lowest EC (0.503 dS/m) was in treatment comprised with 5 t/ha FYM with 2.5 t/ha PM over control (0.570 dS/m). Overall, the combined and alone treatment of FYM showed higher potential to reduce the salt ion concentration in soil as compared to PM. Saline soils, though inherently fertile, are characterized by excessive salt levels that hinder the mineralization of plant nutrients. Research has shown that incorporating crop residues into saline soils enhances nutrient availability to plants and helps regulate soil pH [20]. Effective management of crop residues significantly enhances the soil’s cation exchange capacity (CEC). The application of 10 t/ha of organic substances (biochar alone or combined with FYM) enhanced plant nutrient availability and soil microbial activity in saline soil by regulating soil pH [34]. Residual crop materials enriched with soil organic matter (SOM) can contribute to increased negative charges, thereby elevating the CEC [35]. Crop residues have the potential to reduce salt buildup in superficial soil layers, improve the effectiveness of salt leaching, and increase the soil’s capacity to retain water [36].

### Effect on organic carbon

**Fig 1:**
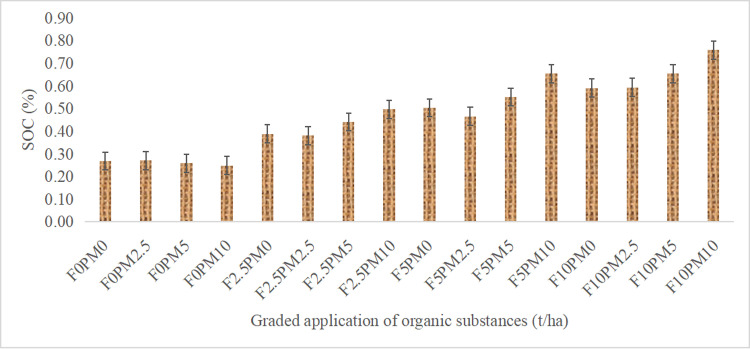
Effect of graded application of FYM and PM on mean SOC.

Organic C is a important parameters under the sustainable soil health management options. Most of the cases addition of organic matter is recommended to enhance the microbial activities for improving mineralization kinetics of organic residues. In this experiment, organic matter more than 10 t/ha was added through FYM and PM combinations under saline soils. The analytical results showed that addition of organic matter either FYM or PM significantly improved the soil organic carbon after a year (Table 1; Fig 1). Periodic soil analysis reported that addition of 20 t/ha (equal part of FYM and PM) improved organic C 0.85% from 0.32% at 3 month time interval. It was observed more than 165% increment compared to control. However, similar trends were observed in most of the time periods except minor reduction in value. It might be due to loss of C during the mineralization process by soil biota. Addition of FYM at 2.5, 5 and 10 t/ha t/ha reported 0.34, 0.45, 0.51% over control (0.22%) after one year incubation period. Similar way of calculation PM application also improved the organic C in lower rate than FYM. Combined application of FYM and PM were improved significantly (p <  0.05) in most of the treatments.

**Table 1:** Effect of graded application of FYM and pressmud on physico-chemical soil proprieties (n = 3).

Treatment	pH				EC (dS/m)				OC (%)			
	Months				Months				Months			
	3	6	9	12	3	6	9	12	3	6	9	12
F_0_PM_0_	8.32	8.22	8.20	8.24	0.573	0.567	0.560	0.570	0.32	0.29	0.24	0.22
F_0_PM_2.5_	8.33	8.23	8.21	8.19	0.673	0.667	0.663	0.650	0.31	0.29	0.24	0.24
F_0_PM_5_	8.24	8.24	8.23	8.22	0.720	0.713	0.703	0.687	0.35	0.26	0.21	0.21
F_0_PM_10_	8.45	8.29	8.28	8.20	0.827	0.817	0.810	0.807	0.38	0.24	0.19	0.18
F_2.5_PM_0_	8.16	7.90	7.89	7.83	0.527	0.523	0.510	0.513	0.45	0.40	0.36	0.34
F_2.5_PM_2.5_	8.24	7.93	7.92	7.90	0.517	0.510	0.507	0.507	0.44	0.39	0.35	0.34
F_2.5_PM_5_	8.17	7.92	7.92	7.90	0.533	0.523	0.517	0.517	0.49	0.44	0.40	0.43
F_2.5_PM_10_	8.25	7.95	7.95	7.93	0.547	0.547	0.540	0.520	0.55	0.50	0.46	0.47
F_5_PM_0_	8.21	7.89	7.86	7.84	0.523	0.537	0.530	0.513	0.57	0.53	0.46	0.45
F_5_PM_2.5_	8.15	7.92	7.91	7.91	0.533	0.520	0.513	0.503	0.53	0.49	0.43	0.41
F_5_PM_5_	8.17	7.90	7.88	7.87	0.543	0.550	0.533	0.530	0.61	0.57	0.51	0.51
F_5_PM_10_	8.34	7.88	7.87	7.87	0.530	0.513	0.513	0.513	0.72	0.68	0.62	0.59
F_10_PM_0_	8.00	7.75	7.75	7.73	0.637	0.630	0.620	0.617	0.70	0.63	0.52	0.51
F_10_PM_2.5_	7.94	7.80	7.81	7.79	0.627	0.617	0.607	0.593	0.66	0.60	0.54	0.57
F_10_PM_5_	8.14	7.89	7.89	7.87	0.627	0.620	0.613	0.613	0.74	0.67	0.58	0.62
F_10_PM_10_	8.20	7.94	7.89	7.81	0.717	0.703	0.677	0.643	0.85	0.76	0.69	0.73
Lsd (p < 0.05)	NS	0.06	0.06	0.07	0.032	0.031	0.050	0.071	0.08	0.07	0.05	0.05

F, Farm yard manure; PM, pressmud.

Microorganisms have the ability to break down crop wastes, which are rich in nutrients [20]. Leftover crop residues comprise a carbon-rich biomass containing essential microelements, with nitrogen levels ranging from 0.6% to 1%, phosphorus from 0.45% to 2%, potassium from 14% to 23%, and carbon comprising 40% to 45%, all crucial for supporting crop growth [37]. Crop residues, abundant in carbon and teeming with microelements such as P, K, and N offer an eco-friendly means of enriching soil quality without disrupting its biological equilibrium. Incorporating crop residues encourages the elevation of organic carbon levels and the availability of essential nutrients like K and P through decomposition, thus providing a nutrient source for both crops and microbial communities [20]. Pressmud can serve as a valuable source of organic matter, offer an alternative method for obtaining crop nutrients, and contribute to soil enhancement [38,39]. Soil organic carbon (SOC) plays a crucial role in influencing the availability and mobility of nutrients for plant uptake. Applied organic substances produced low molecular organic acids during decomposition improved the soil health parameters [40]. Dissolved organic carbon (DOC), on the other hand, acts as an easily degradable substrate that supports microbial activity. The enzyme dehydrogenase, found in living microbial cells, reflects the overall oxidative activity of soil microflora and facilitates the transformation of various nutrients in the soil [41]. Research indicates that the application of manures enhances SOC levels, as they contribute both humified and labile carbon forms from degraded organic matter [24,42].

### Effect on plant nutrients

Available N content in soil was measured at four different time intervals (3, 6, 9 and 12 months). It was observed that the initial analysis data indicated that sole application PM enhanced available N 219 kg/ha (highest applied PM) from 165 kg/ha in control at 3 month time interval (Table 2). However, sole application of FYM increased 177, 200, and 217 kg/ha by the application of 2.5, 5 and10 t/ha over control (no application). The intermixing application of FYM and PM significantly (p <  0.05) improved the mineralization rate and available N in soil, FYM rate 2.5 t/ha with increasing level of PM 2.5, 5 and10 t/ha improved 193, 201, 227 kg/ha. Similar pattern FYM application rate 5 t/ha with PM 2.5, 5 and10 t/ha improved 209, 224, 238 kg/ha and FYM level 10 t/ha with PM 2.5, 5 and10 t/ha improved 226, 233 and 250 kg/ha. Available N after six months incubation reported higher amount of the value ranged 176 kg/ha in control and 262 kg/ha in highest organic residues applied treatments comprised with FYM and PM (equal amount). However, the combination of organic residues level with FYM and PM measured higher amount of available N than sole application of FYM and PM levels. Similar pattern was also observed at 9 month and 12 months time interval. Nitrogen mineralization kinetics was more reported at 12 months time interval. Among the different time intervals, after one year incubation available N was reported more than rest of the periods. It was 69 percent more recovery of available N compared to highest level of organic residues (F_10_ PM _10_). Significant amount of N in FYM and PM enhanced the available N in soil. They help offset the limitations of inorganic fertilizers and mitigate nutrient imbalances in agricultural soil [34]. The properties of crop residues, such as their carbon-to-nitrogen ratio and chemical makeup, along with climatic factors like temperature and moisture, soil conditions like pH and moisture levels, and the method of applying crop residues to the soil [43], collectively influence the rate and extent of nutrient release [44].

**Table 2:** Effect of graded application of FYM and pressmud on soil fertility parameters (n = 3).

Treatment	Available N (kg/ha)				Available P (kg/ha)				Available K (kg/ha)				Available S (kg/ha)			
	Months				Months				Months				Months			
	3	6	9	12	3	6	9	12	3	6	9	12	3	6	9	12
F_0_PM_0_	165	176	207	164	17.90	18.28	18.71	17.98	207	206	207	205	16.6	16.81	17.12	16.55
F_0_PM_2.5_	172	175	212	171	18.41	18.76	18.81	18.73	209	211	212	211	17.1	17.36	17.66	17.67
F_0_PM_5_	191	191	215	197	18.44	18.94	19.32	19.32	213	216	215	210	18.2	18.43	18.74	19.30
F_0_PM_10_	219	225	220	221	19.64	20.17	20.20	20.56	236	241	220	234	18.0	18.25	18.56	19.15
F_2.5_PM_0_	177	178	224	185	18.86	19.21	19.89	20.34	210	208	224	228	16.8	17.15	17.55	17.69
F_2.5_PM_2.5_	193	193	218	204	18.90	19.89	20.57	20.71	209	213	218	217	17.4	17.69	18.09	18.32
F_2.5_PM_5_	201	204	219	214	20.70	22.32	23.00	24.12	220	220	219	223	18.5	18.77	19.16	19.73
F_2.5_PM_10_	227	230	243	245	21.99	22.76	23.44	24.47	237	342	243	245	18.3	18.59	18.99	20.41
F_5_PM_0_	200	207	219	226	18.19	20.39	21.85	21.92	210	217	219	214	18.6	18.90	19.34	19.32
F_5_PM_2.5_	209	213	225	223	18.66	20.13	20.23	22.17	214	221	225	221	18.6	18.96	19.43	19.70
F_5_PM_5_	224	230	253	242	18.77	20.88	21.03	22.29	241	243	253	254	18.9	19.29	19.73	20.27
F_5_PM_10_	238	242	261	257	20.04	20.80	21.81	23.40	249	259	261	262	20.0	20.38	20.82	21.33
F_10_PM_0_	217	231	219	242	19.28	24.04	22.80	23.13	229	238	219	221	18.5	18.94	19.48	19.67
F_10_PM_2.5_	226	235	223	262	19.51	23.81	24.96	25.37	236	267	223	227	18.9	19.41	20.04	20.15
F_10_PM_5_	233	248	271	263	21.35	24.30	26.15	27.13	244	269	271	278	19.0	19.53	20.16	21.09
F_10_PM_10_	250	262	274	277	22.59	26.86	28.97	30.29	260	272	274	287	20.4	20.89	21.93	22.57
Lsd (p < 0.05)	29	30	10	10	1.04	2.08	1.57	1.43	11	6	10	10	0.60	0.62	0.83	0.79

F, Farm yard manure; PM, pressmud.

Available P is a one of the limiting factor in saline soils. However, the addition of organic carbon through the different sources improved the soil microbial diversity and count lead to higher available P recovery. In this experiment, phosphorus level was measured 17.9 in control and highest (22.59 kg/ha) reported after 3 month time interval in FYM_10_PM_10_ treatment (Table 2). Addition of FYM and PM were improved the available P concentration in soil. With increasing the incubation period and organic residues application rate both are enhanced the P availability. Highest FYM and FM applied treated soils showed 22.59, 26.86, 28.97 and 30.29 kg/ha at 3, 6, 9 and 12 month time interval. Among the FYM application rate 0, 2.5, 5 and10 t/ha showed 17.98, 20.34, 21.92, 23.13 kg/ha after 12 months time interval whereas PM applied soils 17.98, 18.73, 19.32, 20.56 kg/ha. It was also observed that FYM treated soils showed better recovery of available P than PM applied levels. Over a span of 30 years, incorporating crop straw into the soil increased the availability of P within the upper 0–20 cm layer. Simultaneously, phosphorus utilization efficiency showed improvement, both with mineral fertilization and with the application of 3750 kg/ha wheat straw treatment, climbing from 43% in 1983 to 72% in 2012 [45].

Available K concentration was measured in this experiment at different time intervals. Increasing the FYM levels from control to 2.5, 5 and 10 t/ha enhanced the 210, 211, 229 kg/ha at 3 months time interval (Table 2); whereas, 208, 217, 238 kg/ha at 6 months; and 224, 219, 219 kg/ha at 9 months and finally 228, 214, 221 kg/ha at 12 months incubation over control (initially 207 kg/ha). Similar way PM residues showed 209, 213, 236 kg/ha at 3 months; 206, 211, 216, 241 kg/ha at 6 months; 212, 215, 220 kg/ha at 9 months; and 211, 210, 234 kg/ha at 12 months time intervals for 2.5, 5 and10 t/ha PM application, respectively. However, mix application of FYM and PM showed better availability of K in soil. The highest recovery was reported in F_10_PM_10_ in all the treatments as 260, 272, 274, 278 kg/ha at 3, 6, 9 and 12 months time intervals. It was also recorded the beneficial effects of organic acids on CEC, speeding up Na^ +^ displacement, and increasing the availability of Ca^2 + ^, Mg^2 + ^, and K^ + ^, all contributing to a reduction in soil sodification [46]. Application of potassic fertilizers with FYM, improved the crop yield and soil health parameters [47]. Pressmud, a solid by-product of sugar mills, is rich in plant nutrients like NPK, organic carbon, and phosphorus [48]. As such, it serves as an excellent organic fertilizer, offering an alternative method for providing crop nutrients and enhancing soil quality.

Available S content in soil was reported during the one year incubation in saline soil. Statistically analyzed data showed the significant difference among the treatments. However, increasing the incubation period significantly (p <  0.05) improved the S concentration in most of the treatments. Highest application rate of FYM and PM (10 t/ha each) showed 20.4, 20.89, 21.93 and 22.57 kg/ha at 3, 6, 9, 12 months time interval (Table 2). Alone application of FYM 2.5, 5, 10 t/ha showed 16.8, 18.6, 18.5 kg/ha at 3 months; 17.15, 18.9, 18.94 kg/ha at 6 months; 17.55, 19.34, 19.48 kg/ha at 9 months; and 17.69, 19.32, 19.67 at 12 months time interval over control, respectively. Similar trend, PM application enhanced the S concentration in saline soil was 17.1, 18.2, 18.0 kg/ha at 3 months, 17.36, 18.43, 18.25 kg/ha at 6 months; 17.66, 18.74, 18.56 kg/ha at 9 months; and 17.67, 19.30, 19.15 kg/ha at 12 months time interval, respectively. FYM treatments showed better availability than PM applied soils. However combination of FYM and PM significantly improved the S concentration 17.4, 18.5, 18.3 kg/ha when FYM @ 2.5 t/ha and PM levels 2.5, 5 and 10 t/ha at 3 months time interval. Similar trends were reported at 6 and 9 month time intervals. However, at 12 months analysis data showed that S concentration was improved by the application of FYM and PM, but the difference was smaller in most of the treatments. Addition of FYM and PM during the experiment mineralized and a significant portion has been released into soil as an available pool. Graded application of bentonite S through FYM improved the S availability and arylsulphatase activities in soils. Mineralization of FYM produced different type of elements act as food substances and improved the soil microbial population and diversity in soil [49]. Over time, incorporating green manuring and applying 2.5 t/ha of gypsum reduced soil pH, boosted the availability of plant nutrients (N, P, K, S, and micronutrients), and improved biological properties (DHA, SMBC) [50,51].

### Effect on soil enzymatic activities

Alkaline phosphatase was analysed at four time interval (3, 6, 9, 12 months) during the incubation period. The FYM and PM addition improved significantly alkaline phosphataes in most of the incubation periods (Table 3). Addition of FYM alone at the rate 2.5, 5 and10 t/ha improved the alkaline phosphatases 18.2, 19.1, 42.3 µg PNP/g soil/h, respectively at 3 months time interval. Similar trend of data analysis showed that PM application were also improved the enzymatic activities 16.0, 20.8, 35.3 µg PNP/g soil/h, respectively at 3 months time interval. The combined application of PM and FYM different levels produced more amount of alkaline phosphatases concentration than the sole application of either FYM or PM. Highest level of FYM (10 t/ha) and PM (10 t/ha) showed 51.8, 63.7, 75.4, 84.0 µg PNP/g soil/h at 3, 6, 9, 12 months time interval, respectively. At 12 month time interval different treatments showed 18.5, 29.8, 43.7, 22.7, 27.2, 35.6, 53.2, 24.1, 31.0, 37.5, 49.0, 58.5, 62.5, 77.4, 84.0 µg PNP/g soil/h in F_0_PM_2.5_, F_0_PM_5_, F_0_PM_10_, F_2.5_PM_0_, F_2.5_PM_2.5_, F_2.5_PM_5_, F_2.5_PM_10_, F_5_PM_0_, F_5_PM_2.5_, F_5_PM_5_, F_5_PM_10_, F_10_PM_0_, F_10_PM_2.5_, F_10_PM_5_, F_10_PM_10_, respectively. Here we can observed that FYM application reported more the concentration than the PM applied soils. Increasing the incubation period also increased the alkaline phosphatase concentration in soil samples. The biomass and growth of soil microorganisms are essential biological indicators for assessing soil health [52]. These biological parameters are very fragile, and greatly affected by environmental factors. According to a review by Rath and Rousk [53], there is no universal pattern linking salinity to soil microbial biomass (or microbial biomass per unit of organic carbon) across different contexts, including natural and modified soils. This variability is due to the various historical and current environmental factors that limit microbial biomass in the soil [54]. Farmers are applying the crop residues for improving the soil fertility level. Two notable effects arise from the utilization of crop residues. First, there are interactions between metals and particulate organic matter originating from crop residues. Second, there is an increase in microbial biomass and enzyme activity subsequent to the application of crop residue [55]. Salt stress negatively impacts plants and soil microbiota by limiting cellular activity, leading to organism mortality [56]. Salinity reduces numerous microbial processes, including respiration, nitrogen mineralization, and enzyme activity [57].

**Table 3:** Effect of graded application of FYM and pressmud on soil microbial properties (n = 3).

Treatment	Alkaline phosphatases (µg PNP/g soil/h)				Arylsulphatase activities (µg PNP/g soil/h)				DHA (µg TPF/g soil/h)			
	Months				Months				Months			
	3	6	9	12	3	6	9	12	3	6	9	12
F_0_PM_0_	14.8	15.0	16.2	15.8	15.6	17.0	16.5	16.2	7.5	7.9	8.3	8.1
F_0_PM_2.5_	16.0	16.3	18.0	18.5	16.7	18.5	19.9	20.0	7.4	7.6	8.1	8.2
F_0_PM_5_	20.8	26.4	29.5	29.8	21.5	21.3	24.2	25.6	8.2	8.2	8.3	8.4
F_0_PM_10_	35.3	41.8	43.6	43.7	36.0	38.1	39.7	40.3	8.3	8.5	8.8	8.8
F_2.5_PM_0_	18.2	20.4	22.3	22.7	17.2	18.6	20.4	20.4	8.1	8.3	8.5	8.5
F_2.5_PM_2.5_	22.8	24.2	26.0	27.2	21.8	22.6	24.9	25.2	8.3	8.6	8.6	8.6
F_2.5_PM_5_	31.6	32.6	34.4	35.6	27.7	28.9	30.8	31.2	8.4	8.6	8.7	8.8
F_2.5_PM_10_	34.2	47.9	49.7	53.2	42.2	43.1	45.4	46.5	8.7	8.9	9.2	9.5
F_5_PM_0_	19.1	21.7	23.9	24.1	19.1	21.2	23.0	23.0	8.4	9.0	10.3	10.5
F_5_PM_2.5_	22.3	26.6	28.4	31.0	21.4	22.4	24.2	25.0	8.4	8.8	11.4	11.6
F_5_PM_5_	27.5	34.1	35.9	37.5	24.9	24.9	26.7	27.1	8.5	8.9	12.0	12.2
F_5_PM_10_	35.8	42.0	43.8	49.0	33.9	34.3	36.1	38.0	9.0	9.5	13.0	13.5
F_10_PM_0_	42.3	44.6	56.4	58.5	43.1	44.3	46.7	46.7	9.9	10.8	13.6	14.1
F_10_PM_2.5_	44.7	47.4	59.1	62.5	43.5	44.3	46.8	48.7	10.4	10.9	14.2	14.6
F_10_PM_5_	51.0	58.8	71.6	77.4	50.2	54.4	56.8	58.8	11.2	11.4	14.8	15.6
F_10_PM_10_	51.8	63.7	75.4	84.0	53.3	54.6	57.0	69.4	12.2	12.6	18.0	19.0
Lsd (p < 0.05)	8.2	1.7	2.1	2.8	1.4	1.5	2.1	2.2	0.45	0.57	0.54	0.67

F, Farm yard manure; PM, pressmud.

Addition of organic substance through also modified the concentration of arylsulphatase activities in soils (Table 3). In this experiments, FYM application rate at 2.5, 5 and10 t/ha improved the concentration by 22.9%, 29.1%, 185.8%, respectively over control at 3 months time interval. Similar pattern, addition of PM also increased the arylsulphatase activities by 8.1%, 40.5%, 138.5% in the treatments 2.5, 5 and10 t/ha, respectively treatments over control. It was also observed that highest combination of FYM and PM different levels were also improved the arylsulphatase activities upto 53.3, 54.6, 57.0, 69.4 µg PNP/g soil/h at 3, 6, 9 and 12 months incubation period, respectively. Higher level of FYM (10 t/ha) highly influenced the arylsulphatase activities when it combined the all levels of the PM. It might be improved the other plant nutrients in soil also. The arylsulphatase activities was reported 20.0, 25.6, 40.3, 20.4, 25.2, 31.2, 46.5, 23.0, 25.0, 27.1, 38.0, 46.7, 48.7, 58.8, 69.4 µg PNP/g soil/h in the treatments F_0_PM_2.5_, F_0_PM_5_, F_0_PM_10_, F_2.5_PM_0_, F_2.5_PM_2.5_, F_2.5_PM_5_, F_2.5_PM_10_, F_5_PM_0_, F_5_PM_2.5_, F_5_PM_5_, F_5_PM_10_, F_10_PM_0_, F_10_PM_2.5_, F_10_PM_5_, F_10_PM_10_, respectively at after 12 months time interval. When FYM was added along with PM, the maximum arylsulfatase activity (ASA) was achieved, measuring 69.4 μg PNP g^ − 1^ soil h^ − 1^. This might be because different organic acids are produced during the breakdown of farmyard manure (FYM), which lowers the soil pH and increases the activity of arylsulfatase in the soil. The F_10_PM_10_ treatment (69.4 μg PNP/ g soil/ h) showed significantly higher ASA in the soil compared to the control (16.2 μg PNP/g soil/h) at 12 months time of incubation period. The sulfate enzyme examined in this experiment, arylsulphatase, is widely distributed in nature [58] and catalyzes the release of SO_4_^2−^ from sulfate esters. Arylsulphatase plays a crucial role in the sulfur cycle and serves as an indicator of sulfur mineralization in soil. Given that arylsulphatase is present both intra- and extracellularly, its activity must be evaluated in both contexts [59]. Many soil enzymes, such as dehydrogenase, urease, alkaline and acid phosphatase, arylsulfatase, and protease, can be inhibited by increased salt levels [60]. In saline soils, the presence of salts impacts plants by changing the pattern of root exudates. To survive under high salinity, soil microorganisms expend energy to maintain osmotic balance, which leads to a reduction in microbial populations and diminishes the rhizosphere’s ability to support root attachment [61].

The DHA is a one of the important microbial parameter which indicated the soil enzymatic activities in soil. It indicates the mineralization kinetics under different organic matter added substances. In this experiment, graded application of FYM (0, 2.5, 5, 10 t/ha) and PM (0, 2.5, 5, 10 t/ha) were applied in different combinations to monitor the effect on DHA. Addition of FYM alone positively improved the DHA level in soil by 8.1, 8.4, 9.9 µg TPF/g soil/h in 2.5, 5, 10 t/ha treatments, respectively at 3 month time interval (Table 3). Similar pattern at 6 month, it was improved 8.3, 9.0, 10.8 µg TPF/g soil/h in 2.5, 5, 10 t/ha treatments, respectively over control treatment. DHA level was also improved at 9 and 12 months time interval. PM application also improved the DHA level in most of the incubation periods 7.4, 8.2, 8.3µg TPF/g soil/h at 3 months; 7.6, 8.2, 8.5µg TPF/g soil/h at 6 months, 8.1, 8.3, 8.8 µg TPF/g soil/h at 9 months; 8.2, 8.4, 8.8 µg TPF/g soil/h at 12 months of incubation period compared to control, respectively. Combined application of FYM and PM in highest applied treatment showed 12.2, 12.6, 18.0 and 19.0 at 3, 6, 9, and 12 months incubation period. During the statistical analysis treatment (F_20_PM_20_) showed 62.7%, 59.5%, 116.7% and 134.6% at 3, 6, 9, 12 months over respective control treatments. Soil enzymes are crucial drivers of soil activity, playing a key role in influencing various physico-chemical properties. They contribute to processes such as organic matter formation, the degradation of xenobiotics, and the availability of essential nutrients like carbon, nitrogen, and phosphorus, all of which are vital for plant growth [62,63]. In this experiment, increasing bioavaialability of plant nutrients also supported by soil enzymatic data. Another notable effect is the increase in microbial biomass and enzyme activity observed after the application of crop residue [64]. The adoption of integrated nutrient management (INM) practices is vital for ensuring long-term crop productivity and maintaining soil health in maize-chickpea cropping systems, particularly in the Vertisols of central India [65]; and in popcirn-potato cropping system [66]. The application of organic amendments plays a critical role in reducing the negative impacts of soil salinity on microbial communities and nutrient cycling processes in paddy rice systems [57]. Su et al. [55] observed that soil treated with corn straw had an increased risk of fungal pathogens and a lower diversity in the fungal community compared to soil receiving wheat straw. Additionally, in cases of double-season straw return, there was a higher relative abundance of actinomycetes, while the relative abundance of bacteria and fungi decreased [55]. It was also observed that the dumping of organic waste on marginal land improved the microbial activities and secretion level of enzymes in soil [67]. Improved level of organic substances in soil, elevated the plant growth-promoting microbes population; and play a crucial role in enhancing crop resilience to environmental stresses, particularly drought and salinity, which are prevalent in arid agro-ecosystems. These beneficial microbes improve plant health through various mechanisms phosphate solubilization, production of growth hormones, and enhancement of root architecture. By fostering better water and nutrient uptake help plants withstand harsh conditions, thereby increasing productivity and sustainability in regions affected by limited water availability and high soil salinity [68]. The integration of agricultural practices offers a sustainable, eco-friendly approach to mitigating the adverse effects of climate change on arid land farming, ensuring food security and ecosystem stability. Kumari et al. [24] found that the addition of various organic inputs enhanced both soil microbial diversity and enzymatic activities. The study also determined that applying just organic manures had positive effects on SOC, DOC, MBC content, DHA, APA, and ASA. The order of these effects was FYM>  pressmud>  poultry manure. Soil treated with FYM exhibited increased microbial activity, enhanced root exudation, greater plant growth, and higher levels of labile C and DOC. This is because FYM immediately served as a substrate for soil microorganisms, resulting in higher MBC [69]. This might be because press mud, a byproduct of sugar mills, contains sugar molecules that break down quickly, thereby increasing microbial activity. Additionally, PM has a high concentration of lignocellulosic compounds, which are essential carbon sources for the microbial breakdown of SOM.

## Conclusion

Saline soils are having good soil structure and nutrient availability except excessive amount of salt. To feed the growing population, India needs 400 mt food grain by the year 2050. Research and development engaged to mediated the physico-chemical properties of saline soil to enhance the production potential of crops. Addition of chemical fertilizers for crop production also adding the salt into the soil, however organic substances like FYM and PM improved the soil health parameters. In this experiments, graded application of FYM and PM was applied in saline soil and incubated for a year. The analytical soil data showed that, after one year pH and EC was declined; whereas, available nutrient status of available N, P, K and S. Soil enzymatic activities like alkaline phosphatases, DHA, arylsulphatase activities were also measured and found significant improvement after one year in FYM and PM (each applied 10 t/ha) over control treatments. It was also observed that FYM performed better than PM. Such findings are very much useful for improving the saline soil by addition of organic substances by including FYM and PM.

### Future suggestions

Soil organic matter in Indian soils is generally low, which adversely impacts fertilizer use efficiency during crop growth. The utilization of organic waste to enhance nutrient availability remains a significant area of research in soil science. Long -term experiment will be conducted under field conditions, incorporating varying salt concentrations and gradients of organic carbon through organic waste. The outcomes are expected to provide a promising solution for improving soil health parameters in areas affected by salinity.

## References

[1] FAO. FAO news world agriculture 2030: main findings. 2021 [cited 2023 June 20]. Available from: https://www.fao.org/english/newsroom/news/2002/7833-en.html.

[2] Bajwa MS. Soil salinity and alkalinity. In: Sekhon GS, Chhonkar PK, Das DK, Goswami NN, Narayanasamy G, Poonia SR, Rattan RK, Sehgal J, editors. Fundamentals of soil science. ISSS; 2002. p. 291–308.

[3] Li D, Qiu H, Tian G, Zhao Y, Zhou X, He S. Soil salinity is the main factor influencing the soil bacterial community assembly process under long-term drip irrigation in Xinjiang, China. Front Microbiol. 2023;14:1291962. doi: 10.3389/fmicb.2023.1291962 38029139 PMC10644797

[4] Dotaniya ML, Meena VD, Saha JK, Dotaniya CK, Mahmoud AED, Meena BL, et al. Use of poor quality water for sustainable crop production in changing scenario of climate change. Environ Develop Sustain. 2022 doi: 10.1007/s10668-022-02365-9 PMC9128324 35645606

[5] Wang C-F, Han G-L, Yang Z-R, Li Y-X, Wang B-S. Plant salinity sensors: current understanding and future directions. Front Plant Sci. 2022;13:859224. doi: 10.3389/fpls.2022.859224 35463402 PMC9022007

[6] Hasanuzzaman M, Raihan MRH, Masud AAC, Rahman K, Nowroz F, Rahman M, et al. Regulation of reactive oxygen species and antioxidant defense in plants under salinity. Int J Mol Sci. 2021;22(17):9326. doi: 10.3390/ijms22179326 34502233 PMC8430727

[7] Liu Y, Xun W, Chen L, Xu Z, Zhang N, Feng H, et al. Rhizosphere microbes enhance plant salt tolerance: toward crop production in saline soil. Comput Struct Biotechnol J. 2022;20:6543–51. doi: 10.1016/j.csbj.2022.11.046 36467579 PMC9712829

[8] Zhang WW, Wang C, Xue R, Wang LJ. Effects of salinity on the soil microbial community and soil fertility. Journal of Integr Agric. 2019;18:1360–8.

[9] Kumar P, Sharma PK. Soil salinity and food security in India. Front Sustain Food Syst. 2020;4:533781. doi: 10.3389/fsufs.2020.533781

[10] Dotaniya ML, Meena MD, Choudhary RL, Meena MK, Singh H, Dotaniya CK, et al. Management of plant nutrient dynamics under alkaline soils through graded application of pressmud and gypsum. PLoS One. 2023;18(8):e0288784. doi: 10.1371/journal.pone.0288784 37556422 PMC10411785

[11] Wu S, Fu W, Rillig MC, Chen B, Zhu Y, Huang L. Soil organic matter dynamics mediated by arbuscular mycorrhizal fungi - an updated conceptual framework. New Phytol. 2024;242:1417–25. doi: 10.1111/nph.19178 37529867

[12] Dotaniya ML, Datta SC, Biswas DR, Dotaniya CK, Meena BL, Rajendiran S, et al. Use of sugarcane industrial byproducts for improving sugarcane productivity and soil health - a review. Int J Recycl Org Waste Agricult. 2016;5(3):185–194 doi: 10.1007/s40093-016-0132-8

[13] Kumar V, Chopra AK. Effects of sugarcane pressmud on agronomical characteristics of hybrid cultivar of eggplant (*Solanum melongena* L.) under field conditions. Int J Recycl Org Waste Agricult. 2016;5:149–62. doi: 10.1007/s40093-016-0125-7

[14] Salman M, Inamullah Jamal A, Mihoub A, Saeed MF, Radicetti E, Ahmad I, et al. Composting sugarcane filter mud with different sources differently benefits sweet maize. Agron. 2023;13(3):748. doi: 10.3390/agronomy13030748

[15] Kumar V, Chopra AK. Ferti-irrigational response of hybrid cultivar of Indian mustard (*Brassica juncea* L.) to distillery effluent in two seasons. Anal Chem Lett. 2013;4(3):190–206.

[16] Bhosale PR, Chonde SG, Nakade DB, Raut PD. Studies on physico-chemical characteristics of waxed and dewaxed pressmud and its effect on water holding capacity of soil. ISCA J Biol Sci. 2012;1(1):35–41.

[17] Brichi L, Fernandes JVM, Silva BM, Vizú J de F, Junior JNG, Cherubin MR. Organic residues and their impact on soil health, crop production and sustainable agriculture: a review including bibliographic analysis. Soil Use Manage. 2023;39:686–706. doi: 10.1111/sum.12892

[18] Saha JK, Rajendiran S, Coumar MV, Dotaniya ML, Kundu S, Patra AK. Soil pollution - an emerging threat to agriculture. Singapore: Springer; 2017. p. 380. doi: 10.1007/978-981-10-4274-4

[19] Meena AL, Jha P, Dotaniya ML, Kumar B, Meena BP, Jat RL. Carbon, nitrogen and phosphorus mineralization as influenced by type of organic residues and soil contact variation in vertisol of central India. Agric Res. 2019;9:232–40. doi: 10.1007/s40003-019-00425-7

[20] Fu B, Chen L, Huang H, Qu P, Wei Z. Impacts of crop residues on soil health: a review. Environ Polluti Bioavailab. 2021;33(1):164–73.

[21] Lahori AH, Tunio M, Ahmed SR, Mierzwa-Hersztek M, Vambol V, Afzal A, et al. Role of pressmud compost for reducing toxic metals availability and improving plant growth in polluted soil: challenges and recommendations. Sci Total Environ. 2024;951:175493. doi: 10.1016/j.scitotenv.2024.175493 39142404

[22] Song X, Sun R, Chen W, Wang M. Effects of surface straw mulching and buried straw layer on soil water content and salinity dynamics in saline soils. Can J Soil Sci. 2020;100(1):58–68. doi: 10.1139/cjss-2019-0038

[23] Leogrande R, Vitti C. Use of organic amendments to reclaim saline and sodic soils: a review. Arid Land Res Manag. 2018;33(1):1–21. doi: 10.1080/15324982.2018.1498038

[24] Kumari M, Sheoran S, Prakash D, Yadav DB, Yadav PK, Jat MK, et al. Long-term application of organic manures and chemical fertilizers improve the organic carbon and microbiological properties of soil under pearl millet-wheat cropping system in North-Western India. Heliyon. 2024;10(3):e25333. doi: 10.1016/j.heliyon.2024.e25333 38333858 PMC10850899

[25] Singh D, Chhonkar PK, Dwivedi BS. Manual on soil, plant and water analysis. New Delhi: Westville; 2005

[26] Subbiah BV, Asija GI. A rapid procedure for the determination of available nitrogen in soils. Curr Sci. 1956;25:259–62.

[27] Olsen S, Cole C, Watanable F, Dean L. Estimation of available phosphorus in soils by extraction with sodium bicarbonate. USDA Circ. 1954;9398:1–19.

[28] Hanway JJ, Heidel H. Soil analyses methods as used in Iowa State College Soil Testing Laboratory. Iowa Agric. 1952;57:1–13.

[29] Williams CH, Steinbergs A. Soil sulphur fractions as chemical indices of available sulphur in some Australian soils. Aust J Agric Res. 1969;10:340–52.

[30] Tabatabai MA, Bremner JM. Use of p-nitrophenyl phosphate for assay of soil phosphatase activity. Soil Biol Biochem. 1969;1(4):301–7. doi: 10.1016/0038-0717(69)90012-1

[31] Casida LE Jr, Klein DA, Santoro T. Soil dehydrogenase activity. Soil Sci. 1964;98(6):371–6. doi: 10.1097/00010694-196412000-00004

[32] Tabatabai MA, Bremner JM. Arylsulfatase activity of soils. Soil Sci Am Proc. 1970;34:225–9. doi: 10.2136/sssaj1970.03615995003400020016x

[33] Gomez KA, Gomez A. Statistical procedures for agricultural research. 2nd edn. New York: Wiley; 1983

[34] Dotaniya ML, Meena MD, Choudhary RL, Meena MK, Meena VD, Singh H, et al. Dynamics of major plant nutrients and enzymatic activities in soil influenced by application of biochar and organic waste. PLoS One. 2024;19(10):e0307487. doi: 10.1371/journal.pone.0307487 39475937 PMC11524486

[35] Rezig FAM, Mubarak AR, Ehadi EA. Impact of organic residues and mineral fertilizer application on soil–crop system: II soil attributes. Arch Agron Soil Sci. 2013;59(9):1245–61. doi: 10.1080/03650340.2012.709623

[36] Dong S, Wan S, Kang Y, Miao J, Li X. Different mulching materials influence the reclamation of saline soil and growth of the *Lycium barbarum* L. under drip-irrigation in saline wasteland in northwest China. Agric Water Manag. 2021;247:10. doi: 10.1016/j.agwat.2020.106730

[37] Wang X, Yang Z, Liu X, Huang G, Xiao W, Han L. The composition characteristics of different crop straw types and their multivariate analysis and comparison. Waste Manag. 2020;110:87–97. doi: 10.1016/j.wasman.2020.05.018 32460108

[38] Bokhtiar S, Paul G, Rashid M, Rahman A. Effect of pressmud and organic nitrogen on soil fertility and yield of sugarcane grown in high Ganges river flood plain soils of Bangladesh. Indian Sugar. 2001;L1:235–40.

[39] Razzaq A. Assessing sugarcane filter cake as crop nutrients and soil health ameliorant. Pakistan Sugar J. 2001;21(3):15–8.

[40] Dotaniya ML, Rajendiran S, Saha JK, Dotaniya CK, Sandeep M. Immobilization of chromium concentration in wheat crop by the application of sugarcane industrial waste. Nat Acad Sci Lett. 2024. doi: 10.1007/s40009-024-01527-9

[41] Meena A, Rao KS. Assessment of soil microbial and enzyme activity in the rhizosphere zone under different land use/cover of a semiarid region, India. Ecol Process. 2021;10(1). doi: 10.1186/s13717-021-00288-3

[42] Liu E, Yan C, Mei X, Zhang Y, Fan T. Long-term effect of manure and fertilizer on soil organic carbon pools in dryland farming in northwest China. PLoS One. 2014;8:e56536. doi: 10.1371/journal.pone.0056536 23437161 PMC3577875

[43] Grzyb A, Wolna-Maruwka A, Niewiadomska A. Environmental factors affecting the mineralization of crop residues. Agron. 2020;10(12):1–18.

[44] Dotaniya ML, Sharma A, Nagar MC, Dotaniya CK, Doutaniya RK, Saha JK. Can application of pressmud mediated plant nutrient dynamics under lead contaminated soils of Indian vertisol? Bull Environ Contam Toxicol. 2023;110(2):44. doi: 10.1007/s00128-023-03690-z 36680693

[45] Guo Z, Liu H, Hua K, Wang D, He C. Long-term straw incorporation benefits the elevation of soil phosphorus availability and use efficiency in the agroecosystem. Span J Agric Res. 2018;16(3):e1101. doi: 10.5424/sjar/2018163-12857

[46] Sheoran P, Kumar A, Singh A. Pressmud alleviates soil sodicity stress in a rice–wheat rotation: effects on soil properties, physiological adaptation and yield-related traits. Land Degrad Dev. 2021;1:1–14.

[47] Kumar S, Dhar S, Barthakur S, Rajawat MVS, Kochewad SA, Kumar S, et al. Farmyard manure as K-fertilizer modulates soil biological activities and yield of wheat using the integrated fertilization approach. Front Environ Sci. 2021;9. doi: 10.3389/fenvs.2021.764489

[48] Dotaniya ML, Datta SC, Biswas DR, Meena HM, Rajendiran S, Meena AL. Phosphorus dynamics mediated by bagasse, press mud and rice straw in inceptisol of north India. Agrochimica. 2015;59(4):358–69.

[49] Dotaniya ML, Meena MD, Choudhary RL, Meena MK, Singh H, Meena VD, et al. Sulphur availability in saline soil mediated by bentonite delivery through FYM. Natl Acad Sci Lett. 2022;45(6):473–5. doi: 10.1007/s40009-022-01137-3

[50] Shirale AO, Kharche VK, Rohi GS, Meena BP. Ameliorative impact of different organic amendments on sodicity and nutrient dynamics in sodic black calcareous soils of Central India. Agrochimica. 2018;LXII(3):219–36.

[51] Shirale AO, Kharche VK, Wakode RR, Meena BP, Das H, Gore RP. Influence of gypsum and organic amendments on soil properties and crop productivity in degraded black soils of central India. Commun Soil Sci Plant Anal. 2018;49(19):2418–28. doi: 10.1080/00103624.2018.1510952

[52] Fierer N, Wood SA, Bueno de Mesquita CP. How microbes can, and cannot, be used to assess soil health. Soil Biol Biochem. 2021;153:108111. doi: 10.1016/j.soilbio.2020.108111

[53] Rath KM, Rousk J. Salt effects on the soil microbial decomposer community and their role in organic carbon cycling: a review. Soil Biol Biochem. 2015;81:108–23. doi: 10.1016/j.soilbio.2014.11.001

[54] Hawkes CV, Keitt TH. Resilience vs. historical contingency in microbial responses to environmental change. Ecol Lett. 2015;18(7):612–25. doi: 10.1111/ele.12451 25950733

[55] Su Y, Yu M, Xi H, Lv J, Ma Z, Kou C, et al. Soil microbial community shifts with long-term of different straw return in wheat-corn rotation system. Sci Rep. 2020;10(1):6360. doi: 10.1038/s41598-020-63409-6 32286481 PMC7156462

[56] Qu W, Li J, Han G, Wu H, Song W, Zhang X. Effect of salinity on the decomposition of soil organic carbon in a tidal wetland. J Soils Sediments. 2018;19(2):609–17. doi: 10.1007/s11368-018-2096-y

[57] Wichern F, Islam MR, Hemkemeyer M, Watson C, Joergensen RG. Organic amendments alleviate salinity effects on soil microorganisms and mineralisation processes in aerobic and anaerobic paddy rice soils. Front Sustain Food Syst. 2020;4.1–14 doi: 10.3389/fsufs.2020.00030

[58] Elsgaard L, Andersen GH, Eriksen J. Measurement of arylsulphatase activity in agricultural soils using a simplified assay. Soil Biol Biochem. 2002;34(1):79–82. doi: 10.1016/s0038-0717(01)00157-2

[59] Lipińska A, Kucharski J, Wyszkowska J. Activity of arylsulphatase in soil contaminated with polycyclic aromatic hydrocarbons. Water Air Soil Pollut. 2014;225(9):2097. doi: 10.1007/s11270-014-2097-4 25221368 PMC4158175

[60] Singh K. Microbial and enzymes activities of saline and sodic soils. Land Degrad Dev. 2016;27:706–18.

[61] Paul D, Lade H. Plant growth promoting rhizobacteria to improve crop growth in saline soils: a review. Agron Sustain De. 2014;34:737–52.

[62] Boyrahmadi M, Raiesi F. Plant roots and species moderate the salinity effect on microbial respiration, biomass, and enzyme activities in a sandy clay soil. Biol Fertil Soils. 2018;54(4):509–21. doi: 10.1007/s00374-018-1277-6

[63] Dotaniya ML, Aparna K, Dotaniya CK, Singh M, Regar KL. Role of soil enzymes in sustainable crop production. In: Khudus M, et al. editor. Enzymes in food biotechnology. Springer International; 2018. p. 569–89.

[64] Su Y, Kwong RWM, Tang W, Yang Y, Zhong H. Straw return enhances the risks of metals in soil? Ecotoxicol Environ Saf. 2021;207:111201. doi: 10.1016/j.ecoenv.2020.111201 32905933

[65] Meena BP, Biswas AK, Singh M, Chaudhary RS, Singh AB, Das H, et al. Long-term sustaining crop productivity and soil health in maize–chickpea system through integrated nutrient management practices in Vertisols of central India. Field Crops Res. 2019;232:62–76. doi: 10.1016/j.fcr.2018.12.012

[66] Meena BP, Kumar A, Lal B, Meena RL, Shirale AO, Dotaniya ML, et al. Sustainability of popcorn-potato cropping system improves due to organic manure application and its effect on soil health. Potato Res. 2019b;62(3):253–79. doi: 10.1007/s11540-018-9410-3

[67] Dotaniya ML, Dotaniya CK, Kumar K, Yadav RK, Doutaniya RK, Meena HM, et al. Ecosystem services from rehabilitated waste dumpsites. In: Pandey VC, editor. Biodiversity and ecosystem services on post-industrial land. Vol. 1. John Wiley & Sons Ltd; 2024, p. 329–55.

[68] Meena A, Meena JK, Dotaniya ML, Gangadhara K, Meena RP, Meena M, et al. Harnessing the potential of plant growth-promoting microbes for alleviating drought and salinity stress in arid agro-ecosystems. In: Rai AK, Chandra P, Basak N, Sundha P, Yadav RK, editors. Rhizospheric interactions for abiotic stress mitigation. UK: Cambridge Scholars Publishing; 2024. ISBN: 978-1-0364-1512-9.

[69] Mazumdar SP, Kundu DK, Saha AR, Majumdar B, Saha R, Singh AK, et al. Carbon and nutrient dynamics under long-term nutrient management in tropical rice-wheat-jute system. Arch Agron Soil Sci. 2018;64(11):1595–607. doi: 10.1080/03650340.2018.1446521

